# Drug Nanocrystal-Loaded Thermo-Reversible Hydrogels of Dexamethasone Palmitate for Intratympanic Drug Delivery

**DOI:** 10.3390/jfb17070347

**Published:** 2026-07-17

**Authors:** Su Yeon Noh, Jee Hyun Kang, In Gyu Yang, Min Young Jeong, Seung Hwan Shin, Jiwon Lee, Hye Ryeong Yoon, Woo Jae Lee, Subin Kim, Seong Su Won, Keum-Jin Yang, Yong Seok Choi, Dong-Kee Kim, Myung Joo Kang

**Affiliations:** 1 College of Pharmacy, Dankook University, 119 Dandae-ro, Dongnam-gu, Cheonan 31116, Republic of Korea; suyeon8540@naver.com (S.Y.N.); dhakflzk@naver.com (I.G.Y.); jmy951207@naver.com (M.Y.J.); sinsung0622@gmail.com (S.H.S.); dlwldnjs8930@naver.com (J.L.); yhr3216@naver.com (H.R.Y.); uzezzang@naver.com (W.J.L.); analysc@dankook.ac.kr (Y.S.C.); 2 Department of Otolaryngology, College of Medicine, The Catholic University of Korea. 64 Daeheung-ro, Jung-gu, Daejeon 34943, Republic of Korea; kangjeehyun@catholic.ac.kr (J.H.K.); blueberry8686@hanmail.net (S.S.W.); nadia@cnu.ac.kr (K.-J.Y.); 3 Department of Otolaryngology, College of Medicine, Soonchunhyang University College of Medicine, Cheonan 31151, Republic of Korea; hgsih12@naver.com

**Keywords:** dexamethasone palmitate, drug nanocrystals, Poloxamer 407, thermosensitive hydrogel, intratympanic delivery, cochlear delivery, cytotoxicity

## Abstract

Intratympanic (IT) injection of corticosteroids is a standard clinical treatment for sensorineural hearing loss; however, achieving both biocompatibility and efficient cochlea drug delivery remains a significant challenge. Herein, we engineered a novel drug nanocrystal (NS)-loaded thermo-reversible hydrogel (NS-TG) system of dexamethasone-21-palmitate (DEX-P), a lipophilic prodrug of dexamethasone (DEX), for improved IT delivery. NSs with a mean diameter of 835.0 nm were fabricated using a dual centrifugation process with polyvinyl alcohol as the stabilizer. The NS-TG system, comprising NSs in a 19% (*w*/*v*) Poloxamer 407 hydrogel, exhibited rapid gelation (2–3 min) and a 31-fold increase in drug solubility (1.57 ± 0.02 mg/mL) compared to NS alone, through micellar solubilization. In vitro cytotoxicity assays in HEI-OC1 cells revealed that NS-TG is highly biocompatible, maintaining cell viability, whereas the commercial lipid emulsion (Lipothason^®^) induced significant cytotoxicity. In vivo pharmacokinetic evaluation in mice revealed that IT NS-TG provided superior cochlear drug absorption compared to DEX-SP solutions and DEX-P NS, despite showing lower absorption than the lipid emulsion. These findings suggest that the DEX-P NS-TG system, pending further investigation, could serve as a promising platform for cochlear steroidal delivery, offering an optimal balance between delivery efficiency and local safety for treating inner ear diseases.

## 1. Introduction

Sensorineural hearing loss (SNHL) is a debilitating auditory impairment characterized by the progressive degeneration of sensory hair cells or auditory nerve fibers within the cochlea [1]. This condition is frequently precipitated by inflammatory processes, acoustic overstimulation, or exposure to ototoxic agents [2]. Although systemic corticosteroid administration remains the clinical gold standard, its therapeutic efficacy is severely constrained by the blood–labyrinth barrier (BLB) [3,4]. The BLB acts as a formidable physiological interface that restricts the entry of systemic pharmacological agents into the inner ear, necessitating high systemic doses that often lead to off-target adverse effects [5]. To circumvent these limitations, intratympanic (IT) injection has emerged as a superior localized delivery route. This approach utilizes the round window membrane (RWM) as the primary portal for drug diffusion into the perilymph, thereby achieving higher intracochlear concentrations while minimizing systemic exposure [5,6,7]. Currently, dexamethasone sodium phosphate (DEX-SP), a hydrophilic prodrug of DEX, is the most prevalent agent for IT therapy due to its excellent aqueous solubility [8,9]. However, its clinical utility is often compromised by two major factors: rapid mucociliary clearance via the Eustachian tube, which reduces residence time within the middle ear cavity, and its inherent hydrophilicity, which limits passive permeability across the lipophilic RWM [9]. The RWM is a complex trilayered anatomical structure comprising an outer epithelium, a middle connective tissue layer, and an inner mesothelium [10,11]. The presence of tight junctions (zonulae occludentes) within these layers produces a charge- and size-dependent barrier, posing a substantial challenge for the diffusion of polar molecules into the inner ear [8,9,10].

To enhance RWM permeability and extend therapeutic duration, lipophilic prodrugs such as dexamethasone-21-palmitate (DEX-P) have gained research interest [12,13]. With an octanol–water partition coefficient (log P) of approximately 9.8, DEX-P exhibits a strong affinity for the lipid bilayers of the RWM, potentially facilitating superior transport compared to hydrophilic analogs [14]. Nevertheless, the extreme lipophilicity of DEX-P necessitates specialized vehicles, typically lipid emulsions (LEs) or liposomes [15,16]. The current commercial benchmark, an LE (Lipothason^®^, Mitsubishi Tanabe Pharma Corp., Osaka, Japan), requires high concentrations of soybean oil (100 mg/mL) and lecithin (12 mg/mL) to solubilize 4 mg/mL of DEX-P [16]. While these excipients are clinically approved for even intravenous use, their administration in the middle ear cavity at such high concentrations raises significant biocompatibility concerns, including the risk of inducing inflammatory responses in the middle ear mucosa or disrupting the sensitive ionic homeostasis and endocochlear potential of the inner ear [17,18].

Drug nanocrystallization technology offers a robust paradigm for the local delivery of poorly water-soluble drugs with minimal excipient requirements, thereby mitigating vehicle-related toxicity. By reducing particle size to the sub-micron range, nanocrystals (NSs) exponentially increase the effective surface area-to-volume ratio, accelerating dissolution velocity as described by the Noyes–Whitney equation and enhancing saturation solubility via the Ostwald–Freundlich effect [19,20,21]. Furthermore, NSs exhibit enhanced mucoadhesiveness; the increased contact area and reduced curvature facilitate stronger van der Waals interactions and physical entanglement with the mucosal surface, potentially extending the drug’s residence time in the cavity [22]. To further improve therapeutic outcomes, the integration of NSs into hydrogel-based systems provides a synergistic delivery platform. Poloxamer 407 (P407), a triblock copolymer (PEO-PPO-PEO), is particularly advantageous due to its unique thermo-responsive rheological properties. P407 undergoes an in situ sol-to-gel transition at physiological temperatures, transforming from a low-viscosity liquid—allowing for ease of injection and optimal spreadability—into a stable, semi-solid drug depot [23]. This depot system effectively sequesters the drug crystals, counteracting rapid Eustachian tube clearance and ensuring prolonged cochlear diffusion [24,25]. Crucially, beyond its gelation capacity, P407 molecules assemble into micellar structures above the critical micelle concentration (CMC) [26]. The hydrophobic poly(propylene oxide) (PPO) cores of these micelles provide an additional microenvironment for the solubilization of lipophilic drugs. This micellar solubilization acts in tandem with the nanocrystal fraction to further enhance the apparent drug solubility.

In the present study, we engineered an NS-loaded thermo-reversible hydrogel (NS-TG) system of DEX-P as an advanced alternative for IT steroidal delivery. DEX-P NSs were fabricated using a dual centrifugation (DC) process and subsequently incorporated into a P407 hydrogel matrix. The concentration of P407 was optimized to ensure the sol-to-gel transition occurs precisely at physiological temperatures. We characterized the physicochemical properties of the NS-TG system, including crystal morphology, particle size, zeta potential, and crystallinity. The rheological behavior, micellar solubilization effects on drug solubility, and in vitro drug release profiles were evaluated. Furthermore, we investigated the in vitro enzymatic conversion of DEX-P to its active form (DEX) and assessed its biocompatibility using the immortalized mouse auditory cell line (HEI-OC1). Finally, the in vivo cochlear absorption of the NS-TG was compared with that of DEX-SP solution and a commercial LE following IT administration in a mouse model.

## 2. Materials and Methods

### 2.1. Materials

DEX-P powder (purity > 98.5%, mean particle size of 20 μm) was provided by Dongkook Pharmaceutical Co. (Seoul, Republic of Korea). Polyoxyethylene (196)–polyoxypropylene (67) glycol (P407), polyoxyl 35 castor oil (Cremophor ELP), macrogol 15 hydroxystearate (Solutol HS15), polyoxyl 40 hydrogenated castor oil (Cremophor RH40), and polyoxyethylene (160)–polyoxypropylene (30) glycol (P188) were provided by CTC Bio (Hwaseong, Republic of Korea). D-(+)-Glucose, polyvinyl alcohol (PVA; average molecular weight of 30,000–70,000 g/mol), sodium chloride, calcium chloride, magnesium chloride, 4-(2-hydroxyethyl)-1-piperazineethanesulfonic acid (HEPES), tyloxapol, sodium carboxymethyl cellulose (Na.CMC; average molecular weight of 90,000 g/mol), hydroxypropyl cellulose (HPC; average molecular weight of approximately 100,000 g/mol), hydroxypropyl methylcellulose (HPMC; number average molecular weight of 86,000 g/mol), polyvinylpyrrolidone K17 (PVP K17), and polyvinylpyrrolidone K30 (PVP K30) were purchased from Sigma-Aldrich (St. Louis, MO, USA). Polysorbate 20 (Tween 20) and polysorbate 80 (Tween 80) were obtained from Croda Korea (Seongnam, Republic of Korea). Potassium chloride and HPLC-grade acetonitrile were purchased from Samchun Chemical Co. (Pyeongtaek, Republic of Korea). High-glucose Dulbecco’s modified Eagle’s medium (DMEM; Cat. No. 11965-092), fetal bovine serum (FBS; Cat. No. SH30919.03), and recombinant mouse interferon-γ (IFN-γ; Cat. No. PMC4034), used for HEI-OC1 cell culture, were obtained from Gibco, a Thermo Fisher Scientific brand (Grand Island, NY, USA), Cytiva HyClone (Logan, UT, USA), and Thermo Fisher Scientific (Waltham, MA, USA), respectively. The EZ-CytoX cell viability assay kit, for cytotoxicity analysis, was purchased from Daeil Lab Service (Seoul, Republic of Korea). Propidium iodide (PI) solution (1.0 mg/mL in water; Cat. No. P3566), for flow cytometric analysis of dead cells, was obtained from Invitrogen, a Thermo Fisher Scientific brand (Carlsbad, CA, USA).

### 2.2. Preparation of DEX-P Loaded NSs Using Bead-Milling Technology

DEX-P-loaded NSs were prepared using a DC-based bead-milling technique, an advanced laboratory-scale method for efficient drug particle size reduction [27,28]. Initially, an isotonic aqueous vehicle was prepared by dissolving 5% (*w*/*v*) glucose in 10 mM phosphate buffer (pH 7.0). Various stabilizers were then dissolved in this vehicle at concentrations ranging from 1 to 10 mg/mL under constant magnetic stirring at 25 °C. To fabricate the NS, 1 mL of the stabilizer solution was transferred into a 2 mL milling tube. Subsequently, 4 or 8 mg of DEX-P powder (4 NS and 8 NS, respectively) and 250 mg of zirconia beads (0.3 mm diameter) were added to the vehicle. The mixture was pre-wetted for 5 min using a vortex shaker at room temperature. The resulting coarse dispersion was subjected to bead milling using a ZentriMix 380R dual-centrifuge (Andreas Hettich GmbH & Co. KG, Tuttlingen, Germany). The milling process was conducted for 1 h at various rotational speeds (500, 1500, and 2500 rpm), while the chamber temperature was maintained at 20 °C throughout the process. Following the milling process, the NS was separated from the zirconia beads using a 26-gauge syringe and collected in a scintillation vial at 25 °C.

### 2.3. Preparation of DEX-P NS-TGs with P407

Different amounts of P407 (160–190 mg) were added to 1 mL of the NS and mixed vigorously by vortexing for approximately 30 min to ensure uniform dispersion. The mixture was then subjected to continuous stirring at 500 rpm using a Twister (VS-96TW, Vision Scientific Co., Ltd., Seoul, Republic of Korea) for 10 h at 4 °C to facilitate complete dissolution of P407 and the formation of a homogeneous solution. The resulting NS-TGs were stored at 4 °C until further experiments.

### 2.4. Physicochemical Characterization of DEX-P NS and NS-TG

#### 2.4.1. Morphology of DEX-P Nanocrystals

The morphology of raw DEX-P material and nanocrystals dispersed in the vehicle or hydrogel was examined using field emission scanning electron microscopy (FE-SEM, MIRA3-LMU, TESCAN, Brno, Czechia). To remove P407, 4 mL of the NS-TG sample diluted 10-fold with vehicle was centrifuged at 13,000 rpm for 10 min (Optima L-100K, Beckman Coulter, Brea, CA, USA). The supernatant was discarded, replaced with an equal volume of distilled water, and the pellet was redispersed. Ten microliters of the resulting suspension were deposited onto an aluminum stub with carbon tape and air-dried at room temperature for 24 h. All samples were sputter-coated with platinum at 30 mA for 30 s (Q150T S plus, Quorum Technologies Ltd., Lewes, UK) and observed at an accelerating voltage of 10 kV.

#### 2.4.2. HPLC Analysis of DEX-P

The amount of DEX-P in the formulation and dissolved was determined using high-performance liquid chromatography (HPLC). For total DEX-P quantification, 20 μL of NS suspension was diluted with 980 μL of mobile phase prior to analysis. To assess the dissolved fractions, 1 mL of each NS sample was centrifuged at 13,000 rpm for 10 min, and the supernatant was diluted two-fold in the mobile phase. The amount of DEX-P in the suspended state was calculated by subtracting the dissolved fraction from the total content in the suspension.

HPLC analysis was performed using a Shimadzu LC-20 series system equipped with a C18 column (4.6 mm × 150 mm, 5 μm, Osaka Soda Co., Ltd., Osaka, Japan). For DEX-P analysis, the mobile phase consisted of 100% acetonitrile, with a flow rate of 1.0 mL/min, an injection volume of 20 μL, and a column temperature of 40 °C. DEX-P was detected at 240 nm. The retention time of DEX-P was 4.7 min. The calibration curve of DEX-P in the range from 1 to 200 μg/mL was linear (r^2^ = 1.0, y = 23,691x − 11,815). The limit of detection (LOD) and limit of quantitation (LOQ) of the analysis method were determined to be 0.089 and 0.27 μg/mL, respectively.

#### 2.4.3. Determination of Particle Size and Zeta Potential

The intensity-weighted hydrodynamic diameters and polydispersity indices (PDI) of the DEX-P NS and NS-TG formulations were determined via dynamic light scattering (Zetasizer Nano, Malvern Instruments, Worcestershire, UK). Samples (100 μL) were diluted 10-fold with distilled water and loaded into disposable cuvettes. Measurements were performed at 25 °C with a 4 mW He-Ne laser (633 nm) at a 90° scattering angle, in triplicate (13 runs, 60 s each). For zeta potential measurement, the samples were diluted 10-fold with 10 mM phosphate buffer (pH 7.0) prior to electrophoretic light scattering analysis to reduce turbidity, viscosity, multiple scattering, and conductivity-related artifacts arising from the concentrated NS and gel matrix. The zeta potential of NSs was determined using a Zetasizer Nano-ZS (Malvern Instruments, Worcestershire, UK) at 25 °C. Twenty runs were performed for each measurement (*n* = *3*).

#### 2.4.4. X-Ray Diffraction (XRD) Analysis

NS and NS-TG samples were ultracentrifuged at 13,000 rpm for 10 min to sediment the drug crystals. For NS-TG samples, the supernatant was discarded after centrifugation, replaced with distilled water, and subjected to a washing step. The precipitated drug crystals were collected using a spatula and dried at 25 °C for 24 h. The diffraction pattern of each dried sample (approximately 20 mg) was determined using an X-ray diffractometer (Ultima IV, Rigaku Corporation, Tokyo, Japan) at 25 °C. Measurements were performed over a scanning range of 5–60°, with a step size of 0.02° and a scan speed of 2°/min.

#### 2.4.5. Thermal Analysis

The thermal properties of raw DEX-P and solidified nanocrystals were assessed by differential scanning calorimetry (DSC; Thermo plus EVO 2, Rigaku Corporation, Tokyo, Japan). Nanocrystals were collected by centrifugation and dried as described above. Approximately 2 mg of each solid sample was sealed in an aluminum pan and heated at 10 °C/min under a nitrogen purge (20 mL/min). An empty pan served as the reference.

#### 2.4.6. Osmolality and pH Determination

The osmolality (mOsmol/kg) and pH of DEX-P NS suspensions were measured directly without dilution. For NS-TG samples, measurements were performed after 20-fold dilution. Osmolality and pH were determined using a micro-osmometer (Micro-osmometer 210, Fiske Associates, Norwood, MA, USA) and a pH meter (S220, Mettler-Toledo LLC., Columbus, OH, USA), respectively.

### 2.5. Rheological Analysis of Sol–Gel Transition of NS-TGs

#### 2.5.1. Gelation Time Measurement

In total, 1 mL of each formulation was placed in a 4 mL scintillation vial and immersed in a water bath maintained at 37 °C. The transition from liquid to solid or semi-solid state was visually monitored, and the time at which the formulation completely lost fluidity was recorded as the gelation time. If no gelation occurred within 300 s, the evaluation was terminated.

#### 2.5.2. Gelation Temperature and Viscosity Determination

Gelation temperature and viscosity of NS-TGs as a function of P407 concentration were evaluated using a modular compact rheometer (MCR 302, Anton Paar, Graz, Austria) equipped with a 25 mm parallel plate and a 1 mm gap. Temperature sweep tests were conducted from 0 °C to 50 °C at a heating rate of 3 °C/min, with a frequency of 1 Hz and a constant stress of 12 Pa. The gelation temperature was defined as the point at which the elastic modulus increased sharply. To compare viscosity at 25 °C and 37 °C, frequency sweep tests were performed after equilibrating samples at each temperature for 10 min, measuring viscosity over a frequency range of 10–100 rad/s under a constant stress of 12 Pa.

### 2.6. In Vitro Dissolution Profile of DEX-P Raw Material, NS, and NS-TG

The in vitro dissolution profiles of DEX-P raw material, DEX-P NSs, and DEX-P NS-TG were evaluated under sink conditions. Cremophor RH40 (1.5% *w*/*v*) was added to the dissolution medium to improve the dispersibility and wetting of DEX-P during the dissolution test. DEX-P raw material was directly added to the dissolution medium at a dose of 4 mg, and 1 mL of each NS or NS-TG formulation was added to the dissolution medium. Each formulation was added to 200 mL of dissolution medium maintained at 37 °C and agitated in a shaking incubator (BF-60SIR, Bio Free, Seoul, Republic of Korea). For NS-TGs, samples were pre-gelled by incubating in plastic containers in a 37 °C water bath for 10 min prior to transfer into the dissolution medium. Artificial perilymph fluid, composed of 130 mM NaCl, 4 mM KCl, 1 mM MgCl_2_, 2 mM CaCl_2_, 6 mM HEPES, and an appropriate amount of glucose in distilled water (adjusted to 300 mOsmol/kg, pH 7.3), was used as the dissolution medium [24,29]. At predetermined time points, 1 mL aliquots were withdrawn from the medium and centrifuged at 13,000 rpm for 10 min to remove any insoluble matter. An equal volume of pre-warmed fresh dissolution medium (37 °C) was added to maintain a constant volume throughout the experiment.

### 2.7. In Vitro Enzymatic Conversion Assay

The in vitro metabolic transformation of the lipophilic prodrug DEX-P into its pharmacologically active form, DEX, was evaluated using porcine liver esterase (PLE). DEX-P NS (equivalent to 10 μg/mL of drug) was incubated in a conversion medium consisting of phosphate-buffered saline (PBS, pH 7.4) supplemented with 0.5% (*w*/*v*) Tween 20 to ensure adequate solubilization of the hydrophobic prodrug. The incubation was conducted in a shaking incubator at 37 °C and 75 rpm, both in the presence and absence (negative control) of 5 U/mL PLE. At predetermined time intervals (0.5, 1, 2, 4, 8, 12, 24, 48, 72, and 96 h), 500 μL aliquots were withdrawn and immediately quenched with 500 μL of ice-cold acetonitrile to terminate enzymatic activity. The quenched samples were then centrifuged at 13,000 rpm for 10 min at 4 °C to precipitate proteins and any remaining particles. The concentration of liberated DEX in the supernatant was subsequently quantified via HPLC. Quantitative analysis of DEX was performed using a validated Shimadzu LC-20 series system (Shimadzu, Kyoto, Japan) equipped with an Aegispak C18-L column (4.6 × 150 mm, 5 μm). The mobile phase consisted of an isocratic mixture of water and acetonitrile (60:40 *v*/*v*) containing 0.1% trifluoroacetic acid. The flow rate was maintained at 1.0 mL/min, and the column temperature was controlled at 40 °C. The detection wavelength was set at 240 nm with an injection volume of 20 μL.

### 2.8. In Vitro Cytotoxicity of DEX-P Formulations

The HEI-OC1 cell line, originally established by Dr. Federico Kalinec [30], was kindly provided by Prof. Min-Young Lee (Dankook University, Republic of Korea). HEI-OC1 auditory cells were maintained in high-glucose DMEM supplemented with 10% FBS and 50 U/mL IFN-γ. Cells were cultured under permissive conditions at 33 °C in a humidified atmosphere containing 10% CO_2_. To evaluate the cytotoxicity of DEX-P formulations, cell viability was assessed using both a colorimetric assay and flow cytometric analysis with PI staining. HEI-OC1 cells were seeded into 96-well plates at a density of 0.7 × 10^4^ cells/well and allowed to attach overnight. Cells were then treated with Lipothason^®^ (Mitsubishi Tanabe Pharma Corporation, Osaka, Japan),4 NS, 8 NS, or 4 NS–19%TG formulations for 24 h. After treatment, 10 µL of EZ-CytoX reagent was added directly to each well according to the manufacturer’s instructions, followed by incubation for 2 h at 33 °C. Absorbance was measured at 450 nm using a microplate reader (Infinite^®^, TECAN, Männedorf, Switzerland). Cell viability was expressed as a percentage relative to untreated control cells. For flow cytometric analysis, cells were harvested by trypsinization, washed twice with PBS, and resuspended in PBS containing 1 µg/mL propidium iodide (PI). Cells were incubated for 15 min at room temperature in the dark. PI-positive cells were quantified using a flow cytometer (FACSCanto II, BD Biosciences, San Jose, CA, USA).

### 2.9. Animals and Auditory Brainstem Response Measurements

All animal experiments were conducted in accordance with the National Research Council Guide for the Care and Use of Laboratory Animals and were approved by the Institutional Animal Care and Use Committee of the Clinical Research Institute, The Catholic University of Korea Daejeon St. Mary’s Hospital (approval number: CMCDJ-AP-2024-001; approval date: 30 May 2024). Eight-week-old male C57BL/6 mice (Orient Bio, Seoul, Republic of Korea) were housed under controlled environmental conditions with free access to food and water. Auditory function was evaluated by auditory brainstem response (ABR) testing at 1 and 2 weeks after intratympanic injection of the formulations. Mice were anesthetized prior to testing, and ABRs were recorded using a TDT System III platform (Tucker-Davis Technologies, Gainesville, FL, USA). Needle electrodes were placed subcutaneously at the vertex (active), beneath the pinna of the operated ear (reference), and beneath the contralateral ear (ground). Recordings were obtained from the operated ear using tone-burst stimuli at 8, 16, and 32 kHz, as well as click stimuli. Tone bursts had a total duration of 4 ms with 1-ms rise/fall times. Sound intensity was decreased in 10-dB steps near the threshold. ABR waveforms were analyzed using BioSig RP software (version 4.4.1; Tucker-Davis Technologies), and the hearing threshold was defined as the lowest sound intensity that elicited a reproducible ABR waveform.

### 2.10. In Vivo Pharmacokinetic Evaluation Following IT Administration in Mice

#### 2.10.1. IT Administration of DEX-P Formulations

For the pharmacokinetic study, mice were anesthetized after a 7-day acclimatization period and placed on a temperature-controlled heating pad. IT injections were performed using a 31 G, 1 mL BD insulin syringe. To normalize the administered dose of DEX, the injection volumes were varied: 5 μL for DEX-SP and 10 μL for all DEX-P formulations (LE, 4 NS, 8 NS, and 4 NS–19%TG). Consequently, DEX-SP, LE, 4 NS, and 4 NS–19%TG were administered at an equivalent absolute dose of approximately 25 μg as DEX, whereas 8 NS was administered at approximately 50 μg as DEX.

#### 2.10.2. Determination of DEX in Cochlear Lysates

Cochlear drug concentrations were evaluated at predetermined time points following IT injection of DEX-P solution or DEX-P NS formulations. Cochlear tissues were harvested, rinsed in isotonic PBS to remove surface drug residues, and stored at −80 °C until analysis. Tissues were weighed and pulverized using a pestle, then mixed with 400 μL acetonitrile and shaken for 10 min. After centrifugation at 13,000 rpm, 4 °C for 10 min, 40 μL of the supernatant was combined with 460 μL acetonitrile and 500 μL acetonitrile containing 200 ppb of internal standard (triamcinolone acetonide). The mixture was centrifuged again, and the supernatant was transferred to LC vials for LC-MS/MS analysis. DEX concentrations in cochlear tissue were quantified using a validated liquid chromatography–tandem mass spectrometry (LC-MS/MS) [31]. Chromatographic separation was performed on a Shimadzu Nexera UPLC system (Tokyo, Japan) with a Phenomenex Gemini NX-C18 column (2.1 × 150 mm, 3 μm, Torrance, CA, USA) under gradient elution with 0.1% formic acid in water (A) and 100% acetonitrile (B). The mass spectrometer (LCMS-8050, Shimadzu Corporation, Tokyo, Japan) operated in positive electrospray ionization (ESI) mode using multiple reaction monitoring (MRM). The autosampler and column oven were set at 4 °C and 40 °C, respectively, with a 10 μL injection volume. The interface, DL, and heating block temperatures were set at 300 °C, 250 °C, and 400 °C, respectively.

Pharmacokinetic parameters, including maximum drug cochlear concentration in cochlear homogenates (C_max_), time to reach C_max_ (T_max_), and the area under the drug concentration-time curve up to the last measurable time point (AUC_last_), were determined from the cochlear tissue drug concentration data obtained at 0, 1, 3, 6, and 24 h using WinNonlin software (version 5.2, Pharsight Co., Mountain View, CA, USA). The C_max_, T_max_, and AUC_last_ values were obtained directly from the WinNonlin output.

### 2.11. Lyophilization and Evaluation of Short-Term Storage Stability of DEX-P NS Formulations

To ensure physicochemical stability, the prepared DEX-P NS and NS-TG were subjected to a standardized lyophilization cycle. The aqueous NS formulations were aliquoted into USP Type I glass vials and frozen at −85 °C for 24 h to induce complete solidification. The frozen samples were subsequently dried at −85 °C for 24 h. The lyophilized NS-loaded vials were transferred to a calibrated stability chamber (25 ± 2 °C and 60 ± 5% RH) over a 4-week period. Visual appearance was evaluated for cake shrinkage and matrix collapse. Visual appearance was rigorously monitored for cake shrinkage and matrix collapse. For reconstitution, sterile water for injection was added dropwise to the lyophilized cake, followed by gentle manual agitation to achieve complete redispersion. The drug content, hydrodynamic diameter, PDI, and zeta potential of the reconstituted nanocrystals were determined immediately following the reconstitution process.

### 2.12. Statistical Analysis

All experiments were performed at least three times, and data are expressed as means ± standard deviation (SD). One-way analysis of variance, followed by Tukey’s post hoc test, was used to compare differences between groups. Data analysis was performed using SPSS, version 22.0 (IBM Corp., Armonk, NY, USA). Statistical significance was set at *p* < 0.05.

## 3. Results and Discussion

### 3.1. Design of DEX-P NSs Using Bead-Milling Technique

In the present study, we engineered a DEX-P NS system for intracochlear drug delivery following IT administration. To the best of our knowledge, this is the first report of a solid-state nanocrystal strategy designed for this lipophilic corticosteroid prodrug. Currently, pharmacological interventions for inner ear delivery of DEX-P have predominantly relied on lipid-based solubilization platforms, such as oil-in-water emulsions and phospholipid-based liposomes [15,16]. While these lipidic nano-architectures effectively solubilize the lipid prodrug and facilitate penetration through the RWM, their clinical utility is frequently compromised by biocompatibility concerns within the delicate cochlear environment [17,32]. Commercialized lipid-based carriers typically require high concentrations of corn oils and phospholipids to maintain drug solubility and physical stability [16]. For instance, LE contains high concentrations of soybean oil (100 mg/mL) and lecithin (12 mg/mL) to solubilize 4 mg/mL of DEX-P [16]. Such high surfactant-to-drug ratios can induce ototoxicity, inflammatory responses, and mucosal thickening, potentially damaging hair cells and the stria vascularis. Furthermore, exogenous oily components may disrupt endocochlear fluid dynamics or cause localized edema [17,32]. To address these limitations, we designed a biocompatible NS system using minimal stabilizers, hypothesizing that this approach would preserve cochlear integrity while enhancing delivery through increased surface area and accelerated dissolution kinetics [19,20,33,34].

**Figure 1:**
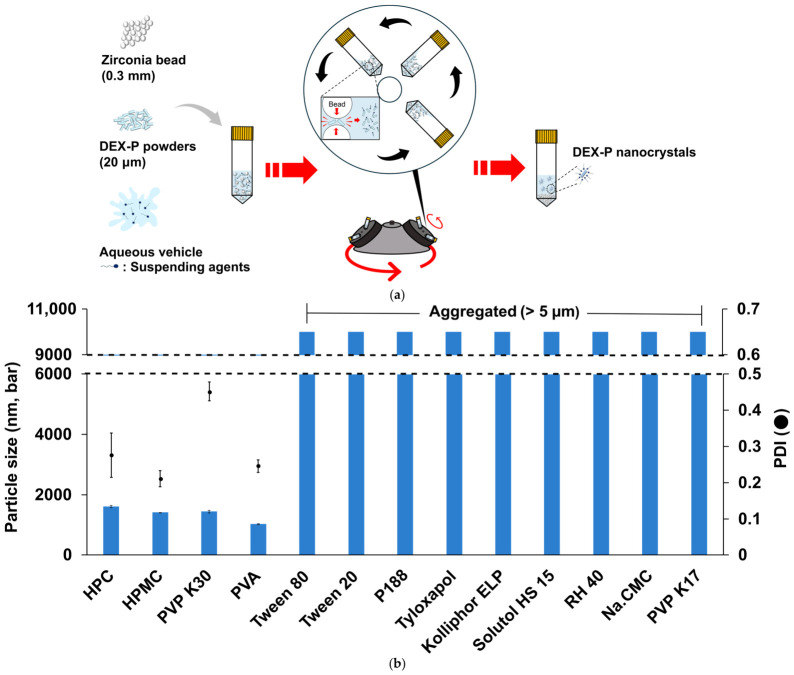

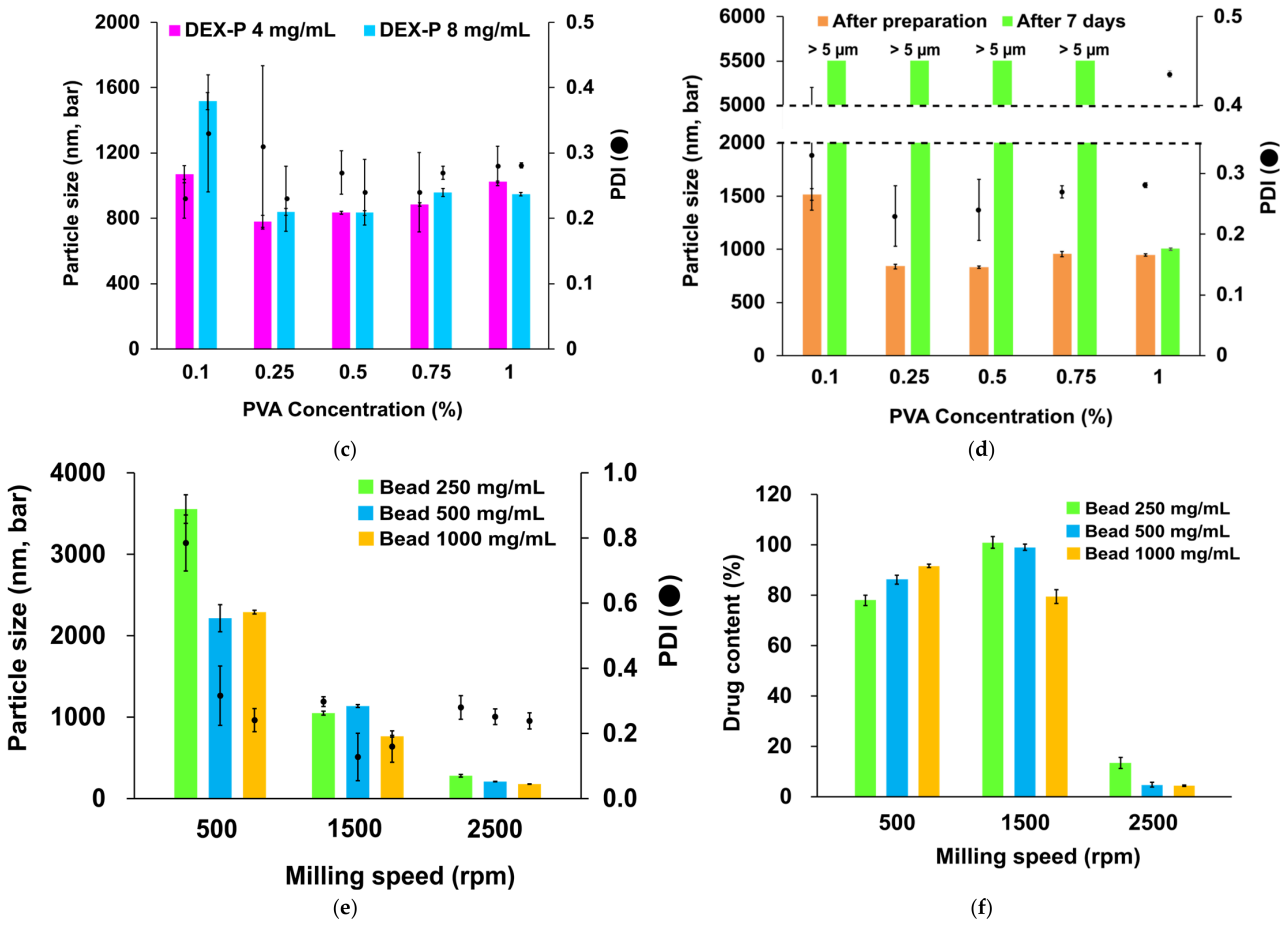
Optimization of fabrication parameters for DEX-P NSs. (**a**) Schematic illustration of the preparation process for DEX-P NSs via the bead-milling process. (**b**) Screening of suspending agents based on their effects on particle size and PDI. (**c**) Influence of PVA concentration (0.1–1%) on particle size and PDI at two different drug loadings (4 mg/mL and 8 mg/mL). (**d**) Particle size of NSs after 7 days of storage at 25 °C as a function of PVA concentration. (**e**) Impact of milling speed (rpm) and bead amount (250–1000 mg) on particle size and PDI. (**f**) Effect of milling speed and bead amount on drug recovery after bead milling. Notes: (**b**) The concentrations of the suspending agent and DEX-P were set to 0.1% and 4 mg/mL, respectively, with a bead amount of 250 mg, a milling speed of 1500 rpm, a milling time of 1 h, and a temperature of 20 °C. (**c**) The milling speed, milling time, temperature, and bead amount were set to 1500 rpm, 1 h, 20 °C, and 250 mg, respectively, with different PVA concentrations. (**d**) The DEX-P concentration, milling speed, milling time, temperature, and bead amount were set to 8 mg/mL, 1500 rpm, 1 h, 20 °C, and 250 mg, respectively, and the samples were stored at 25 °C for 7 days. (**e**,**f**) The DEX-P and PVA concentrations were set to 4 mg/mL and 1%, respectively, with different milling speeds and bead amounts. The milling time and temperature were set to 1 h and 20 °C. (**b**–**f**) Data are presented as mean ± SD (*n* = *3*).

DEX-P NSs were fabricated using a DC technique with zirconia beads as the grinding medium (Figure 1a). Unlike conventional bead milling, this process generates intense mechanical energy through simultaneous rotation and revolution, inducing high-frequency collisions and potent shear forces that facilitate efficient size reduction via mechanical attrition [27,28]. For the highly lipophilic DEX-P, the kinetic energy transferred from the zirconia beads surmounts the crystal lattice energy of the drug, leading to rapid propagation of fractures along its cleavage planes. This energetic comminution effectively breaks down the initial coarse DEX-P particles into sub-micron dimensions by providing high-frequency impact stress that exceeds the fracture limit of the crystalline drug. For clinical relevance, drug loading was set at 4–8 mg/mL with glucose added for isotonicity.

Different surfactants and hydrophilic polymers were screened to ensure the physical stability of the sub-micron particles. As shown in Figure 1b, most stabilizers (e.g., Tween 80, Tween 20, P188, Tyloxapol, PVP K17, Kolliphor ELP, Na.CMC) failed to suppress particle growth, resulting in severe aggregation with dimensions > 5 μm immediately following the DC process. In contrast, the use of hydrophilic polymers such as HPC, HPMC, PVP K30, and PVA successfully yielded stable drug nanocrystals with mean diameters ranging from 900 to 1600 nm. PVA achieved the most significant size reduction to 900 nm while maintaining a narrow size distribution (PDI = 0.246 ± 0.017) among the screened stabilizers. The effectiveness of PVA is attributed to its amphiphilic nature: hydrophobic segments adsorb onto the DEX-P crystal surfaces, while hydrophilic chains extend into the aqueous phase, forming a steric barrier that counteracts van der Waals forces and suppresses Ostwald ripening [35,36].

The effects of PVA concentration (0.1–1.0%) and drug loading (4 and 8 mg/mL) on mean particle size and PDI were further evaluated (Figure 1c,d). A clear concentration-dependent trend was observed; increasing the PVA concentration to 1.0% significantly reduced mean particle size and improved PDI, yielding a PDI of approximately 0.28 for both the 4 NS and 8 NS formulations. This improvement results from enhanced surface coverage, which increases surface pressure and prevents coalescence [35,36]. Notably, the 8 mg/mL DEX-P formulation with 1.0% PVA remained stable without significant growth or sedimentation for 7 days at 25 °C. The relationship between milling intensity (speed and bead volume) and particle dimensions was systematically investigated (Figure 1e). While higher mechanical energy, particularly at the highest milling speed of 2500 rpm, reduced particle size to approximately 200–300 nm, it also caused a critical reduction in drug recovery. At the highest milling intensities, a 90% loss of drug content was observed, leaving only 10% of the initial dose. This dramatic loss is likely due to non-specific adsorption of the highly lipophilic DEX-P (Log P = 9.8) onto the zirconia beads during milling. As particle size decreases, the total surface area increases exponentially, enhancing the drug’s affinity for the grinding medium [28,37]. Such substantial losses present a significant hurdle for precise clinical dosing.

Based on these findings, formulation parameters were adjusted to balance particle size reduction with drug recovery. The final conditions—drug concentration of 4 or 8 mg/mL, PVA concentration of 0.5% for 4 mg/mL (4 NS) and 1.0% for 8 mg/mL (8 NS), and a milling speed of 1500 rpm—produced DEX-P NSs with a reproducible mean particle size of approximately 1000 nm.

### 3.2. Design of DEX-P NS-TGs with P407

To prolong the residence time of DEX-P NS within the middle ear and facilitate sustained drug release, a thermo-responsive in situ gelling system (DEX-P NS-TG) was designed using P407. P407 is a non-ionic surfactant widely recognized for its favorable biocompatibility profile and extensive utilization in clinical applications [38,39]. The clinical viability of such systems is supported by randomized trials of OTO-104, a thermo-sensitive DEX hydrogel, which demonstrated high tolerability and no adverse effects on hearing or vestibular function following IT injection [40].

**Figure 2:**
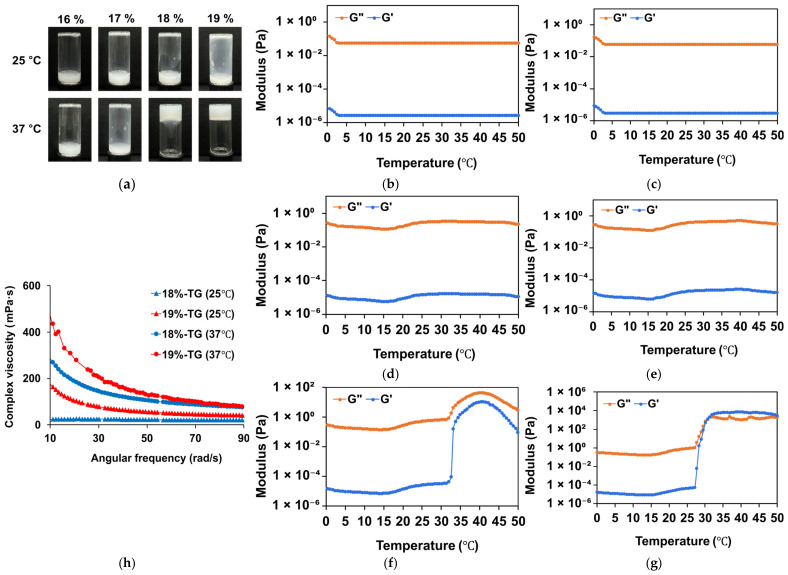
Rheological properties and sol–gel transition behavior of DEX-P NS and NS-TGs. (**a**) Visual characterization of the sol–gel transition for NS-TGs prepared with P407 (16–19 wt%) after 5 min of incubation at 25 °C and 37 °C. (**b**–**g**) Temperature-dependent rheological profiles showing the storage modulus (G′) and loss modulus (G″) of (**b**) 4 NS, (**c**) 8 NS, (**d**) 4 NS–16%TG, (**e**) 4 NS–17%TG, (**f**) 4 NS–18%TG, and (**g**) 4 NS–19%TG. (**h**) Frequency-dependent complex viscosity profiles of 4 NS–18%TG and 4 NS–19%TG as a function of angular frequency. Notes: (**a**) Samples were equilibrated at each temperature for 5 min before visual observation. (**b**–**g**) Temperature sweep analysis was performed from 0 °C to 50 °C at a heating rate of 3 °C/min, with a frequency of 1 Hz and a constant stress of 12 Pa. (**h**) Frequency sweep analysis was performed after sample equilibration at 25 °C or 37 °C for 10 min, and complex viscosity was measured over an angular frequency range of 10–100 rad/s under a constant stress of 12 Pa.

The concentration-dependent phase transition of DEX-P NS-TG was initially evaluated using the tube-inversion method (Figure 2a). At room temperature (25 °C), formulations containing 16–19% (*w*/*v*) P407 maintained a low-viscosity, fluidic sol state. This flowability is clinically advantageous, as it ensures ease of administration through fine-gauge needles during IT procedures [25]. Upon heating to physiological temperature (37 °C), a distinct concentration-dependent gelation was observed. While formulations with 16% and 17% P407 failed to form a stable gel matrix, those with ≥18% P407 underwent a complete transition into a non-flowing semi-solid state within 2–3 min (Figure 2a).

Temperature-dependent viscoelastic properties were further characterized by monitoring the storage modulus (G′) and loss modulus (G″) from 0 to 50 °C (Figure 2b–g). For intact NSs (4 NS and 8 NS), G″ consistently exceeded G′ across the entire temperature range, confirming their predominantly viscous, liquid-like behavior (Figure 2b,c). Formulations with lower P407 concentrations (16% and 17%) exhibited similar rheological profiles with G″ remaining dominant, indicating that micellar density was insufficient to induce a complete sol–gel transition at 37 °C. In contrast, 4 NS–18%TG (Figure 2f) exhibited a sharp increase in both moduli starting at ~30 °C, signifying the onset of micellar entanglement [38]. The most pronounced transition was observed in 4 NS–19%TG (Figure 2g), where both G′ and G″ increased dramatically—by several orders of magnitude—between 20 °C and 25 °C. In this system, the crossover point where G′ exceeded G″—defined as the sol–gel transition temperature (T_sol–gel)—was identified within a range suitable for immediate in vivo gelation upon administration. Increasing P407 concentration shifted T_sol–gel toward lower temperatures, attributable to higher micellar density that promotes closer packing and inter-micellar dehydration at reduced thermal energy [41,42].

The complex viscosity (η*) was further evaluated to characterize the resistance of the formulations to flow and deformation under oscillatory stress [41]. Intact NSs showed negligible η* values across the frequency range, consistent with their Newtonian-like fluid behavior. Conversely, P407-containing formulations exhibited significantly higher η* values and a characteristic shear-thinning profile, where viscosity decreased with increasing angular frequency (Figure 2h). At 25 °C, 4 NS–19%TG showed higher η* than 4 NS–18%TG, reflecting increased flow resistance even at room temperature. At 37 °C, the η* of 4 NS–19%TG remained consistently higher than that of 4 NS–18%TG across all tested frequencies, suggesting the formation of a denser and more mechanically robust micellar network. Based on these rheological findings, 4 NS–19%TG was selected for further studies. Its robust gelation and superior mechanical strength are expected to prevent premature drainage through the Eustachian tube, thereby maximizing the contact time of the nanocrystals with the RWM and enhancing intracochlear drug delivery [25,43].

### 3.3. Physicochemical Characteristics of DEX-P NS and NS-TG

**Figure 3:**
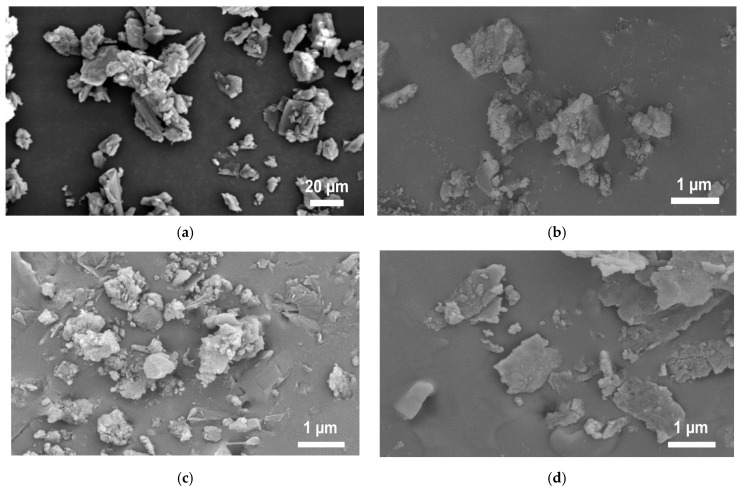
Representative SEM images of (**a**) DEX-P raw material, (**b**) 4 NS, (**c**) 8 NS, and (**d**) 4 NS–19%TG. Note: Scale bars in the SEM images indicate (**a**) 20 µm and (**b**–**d**) 1 µm.

The physicochemical properties of the optimized DEX-P NSs (4 NS, 8 NS) and 4 NS–19%TG were characterized in terms of morphology, particle size, surface charge, crystallinity, drug suspension-to-dissolution ratio, osmolarity, and pH (Table 1). SEM observations revealed a distinct morphological transition from large, irregular bulk DEX-P particles (>20 µm) to well-dispersed nanocrystals in the 4 NS, 8 NS, and 4 NS–19%TG formulations (Figure 3). The resulting nanocrystals exhibited irregular geometries lacking distinct crystalline facets, which may be attributed to the intrinsic crystalline habit of the DEX-P raw material. This reduction in size was quantitatively corroborated by dynamic light scattering analysis, which yielded mean hydrodynamic diameters of 835.0 ± 9.5 nm and 946.3 ± 10.9 nm for 4 NS and 8 NS, respectively. The low PDI values below 0.3 observed in 4 NS and 8 NS indicate a narrow and uniform size distribution (Table 1). Upon incorporation into the P407 matrix, the particle size of 4 NS–19%TG slightly increased to 1176.0 ± 11.0 nm, which is likely attributed to the adsorption of poloxamer polymer chains onto the nanocrystal surfaces [36,38]. SEM observations confirmed that the drug nanocrystals maintained their original particle size without aggregation within the matrix (Figure 3). However, due to the essential pre-treatment involving the dilution and removal of P407 prior to imaging, the three-dimensional porous network structure of the hydrogel matrix was not observed. The zeta potential of all formulations ranged from −4.4 to −11.6 mV, reflecting a relatively neutral to slightly negative surface charge. This near-neutrality is primarily attributed to the non-ionic nature of the core components, including DEX-P, PVA, and P407. Such neutral surface characteristics are expected to minimize potential irritation at the localized administration site upon IT injection [44].

The drug content within the NS systems ranged from 92% to 101% of the target concentration (3.86–7.80 mg/mL), indicating that no significant drug degradation occurred during the fabrication process (Table 1). The distribution of the drug between dissolved and suspended states was significantly influenced by the presence of the P407 polymer. While the dissolved DEX-P concentration in the 4 NS and 8 NS remained negligible (0.05–0.08 mg/mL), the 4 NS–19%TG exhibited a 31-fold increase in apparent solubility, reaching 1.57 ± 0.02 mg/mL. This marked enhancement is primarily attributed to micellar solubilization facilitated by P407 [38,45]. Given that the CMC of P407 is approximately 2.8 × 10^−6^ M (or roughly 0.035% *w*/*v*) at 25 °C, the P407 concentration employed in this formulation (19%) significantly exceeds the CMC [46]. This ensures the spontaneous formation of stable micelles that effectively encapsulate hydrophobic drug molecules within their lipophilic cores [38,45]. Furthermore, the system successfully maintains a substantial nano-suspended fraction (2.32 ± 0.08 mg/mL) of DEX-P, which serves as a reservoir to facilitate a sustained release profile.

**Figure 4:**
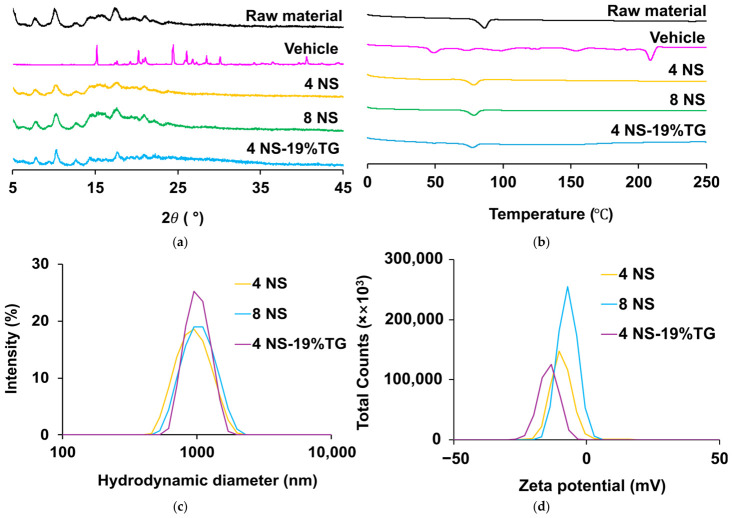
Physical characteristics of DEX-P NS systems. (**a**) XRD patterns (**b**) DSC thermograms of raw material, vehicle, 4 NS, 8 NS, and 4 NS–19%TG. (**c**) Particle size distribution curves, and (**d**) Zeta potential distributions of 4 NS, 8 NS, and 4 NS–19%TG. Note: (**c**) Mean hydrodynamic size determined using dynamic light scattering measurement technology (Zetasizer Nano^®^ Instruments).

Despite the shift in solubility, the crystalline nature of the raw material was largely preserved in all NS formulations. XRD patterns of 4 NS, 8 NS, and 4 NS–19%TG exhibited characteristic diffraction peaks at 2θ angles of approximately 12.3°, 13.5°, 15.1°, and 16.9°, which were identical to those of the raw DEX-P material (Figure 4a). The absence of any significant shift denotes that the crystal lattice of DEX-P remained unaltered during the bead-milling process [24,47]. Furthermore, DSC thermograms revealed an endothermic melting peak at approximately 72–80 °C for all NSs, with a slight downward shift compared to the raw drug (Figure 4b). This minor reduction in the melting point is attributable to the significant reduction in particle size to the nanometer scale—often associated with the Gibbs–Thomson effect—as well as the potential presence of residual excipients interacting with the drug nanocrystals [48]. These results collectively suggest that the DC process effectively produced stable nanocrystals without inducing amorphous transformation or polymorphic changes, and that crystallinity was effectively maintained within the thermos-sensitive gel matrix. All formulations exhibited physiological pH values (7.00–7.07) and isotonic osmolarity (300.0–328.0 mOsmol/kg), ensuring excellent biocompatibility for IT administration.

### 3.4. In Vitro Dissolution Profile of DEX-P NS and NS-TG

**Figure 5:**
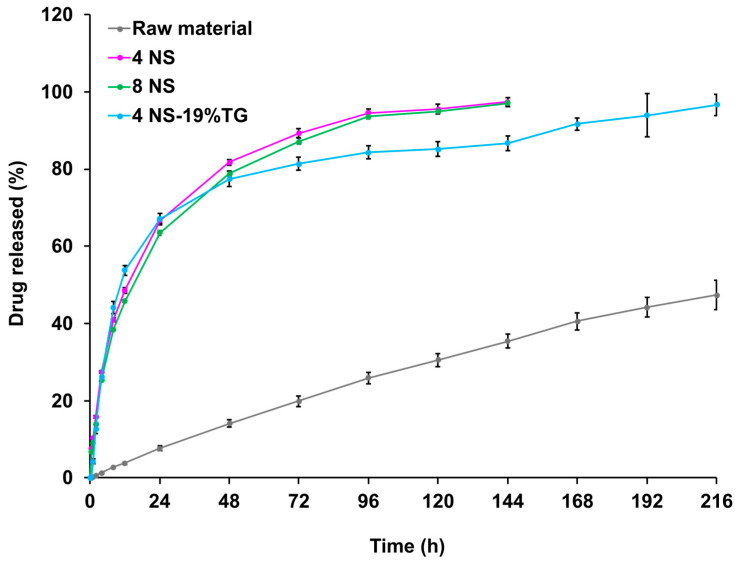
Comparative in vitro dissolution profiles of DEX-P raw material, 4 NS, 8 NS, and 4 NS–19%TG under sink conditions. Notes: The dissolution medium was artificial perilymph fluid (pH 7.3) supplemented with 1.5% (*w*/*v*) Cremophor RH40 to maintain sink conditions for the hydrophobic drug. The data points represent the mean drug release percentage, with error bars indicating the standard deviation of three independent experiments (mean ± SD, *n* = *3*).

The in vitro dissolution profiles of the raw DEX-P, 4 NS, 8 NS, and 4 NS–19%TG were evaluated in artificial perilymph fluid (pH 7.3) to simulate the physiological environment of the inner ear (Figure 5). To ensure sink conditions for the highly lipophilic DEX-P (aqueous solubility < 0.01 mg/mL), the dissolution medium was supplemented with 1.5% (*w*/*v*) Cremophor RH40. Cremophor RH40, a non-ionic solubilizer, effectively enhances the apparent solubility of poorly water-soluble drugs primarily through micellar solubilization. By reducing the interfacial tension between the hydrophobic drug particles and the aqueous medium, it promotes efficient wetting and encapsulates DEX-P molecules within its micellar cores, thereby facilitating a more rapid and consistent dissolution process [45,49].

Under these conditions, the equilibrium solubility of DEX-P was increased to approximately 1.0 mg/mL. Given that 4 mg of the drug was added to 200 mL of the medium, the total volume provided a 50-fold excess compared to the saturation volume, maintaining sink conditions throughout the experiment. Although a low-volume, surfactant-free release setup—ideally integrated with a biomimetic RWM permeation model is highly desirable, establishing such a micro-scale system posed substantial technical challenges. Consequently, a standardized sink condition setup was utilized to evaluate the comparative release kinetics of DEX formulations. No conversion of DEX-P to its hydrolyzed form, DEX, was detected during the dissolution period.

As shown in Figure 5, 4 NS and 8 NS provided a remarkably rapid dissolution profile, reaching approximately 80% cumulative release within the first 48 h, whereas the raw material achieved less than 15% during the same period. The DEX-P raw material exhibited extremely poor dissolution behavior due to its inherent hydrophobicity and low aqueous solubility [14]. The hydrophilic PVA layer on the nanocrystal surfaces effectively reduces the interfacial tension and contact angle between the drug and the dissolution medium, facilitating rapid wetting and dissolution [35,36]. This dramatic improvement is fundamentally attributed to the nano-sizing process, which exponentially increases the effective surface area available for dissolution. According to the Noyes–Whitney equation, the dissolution rate is directly proportional to the surface area of the drug particles [50]; thus, the reduction of DEX-P crystals to the nanometer range maximizes the rate of mass transfer from the solid surface to the bulk medium. Beyond the increase in surface area, the high-energy milling process further contributes to dissolution enhancement by decreasing the crystalline integrity of the drug. The intense mechanical stress applied during milling can induce lattice defects or a partial reduction in the crystallinity index, thereby lowering the lattice energy [20,47]. No significant difference in release kinetics was observed between 4 NS and 8 NS, indicating that the nano-sizing effect and the resulting physicochemical changes remain consistent regardless of the initial drug concentration, provided that a similar particle size distribution is maintained. These findings underscore the efficiency of the NS system in overcoming the dissolution-limited absorption typically associated with highly lipophilic prodrugs like DEX-P.

The NS–TG system displayed a distinct biphasic release profile. During the initial stage (up to 24 h), 4 NS–19%TG exhibited an even faster release rate than 4 NS, reaching over 65% cumulative release (Figure 5). This initial “burst” effect is attributed to the significantly higher fraction of pre-dissolved drug within the 4 NS–19%TG; as detailed in Table 1, the dissolved DEX-P concentration in 4 NS–19%TG (1.57 ± 0.02 mg/mL) was nearly 31-fold higher than that in 4 NS (0.05 ± 0.00 mg/mL) due to the solubilizing effect of P407 [38,45]. However, after reaching approximately 70% release, the release rate of 4 NS–19%TG became notably slower than that of 4 NS. This transition indicates that the hydrogel matrix acts as a diffusion barrier. Once the dissolved drug is rapidly released, the remaining nanocrystals undergo dissolution and subsequent diffusion through the tortuous three-dimensional network of the hydrogel matrix [51,52]. This dual mechanism is expected to provide rapid therapeutic levels followed by prolonged drug exposure within the middle ear cavity.

### 3.5. In Vitro Enzymatic Conversion of DEX-P to DEX

**Figure 6:**
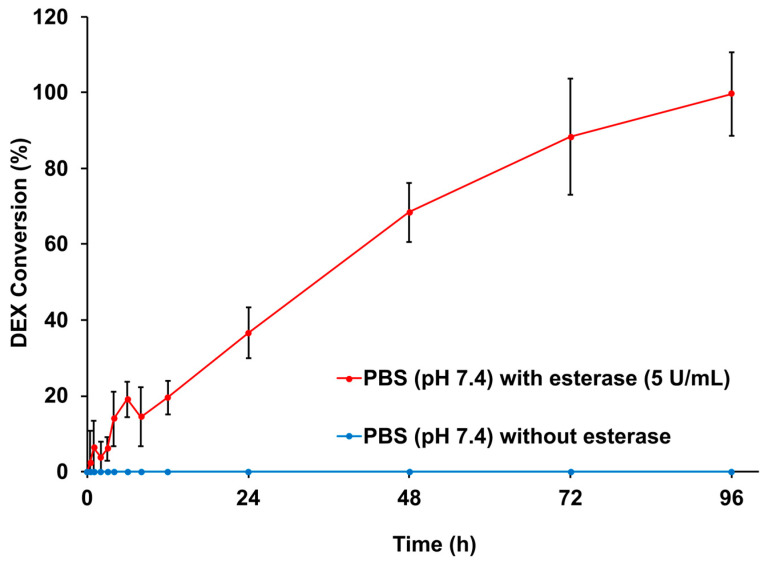
In vitro conversion profile of DEX-P to DEX in the presence (5 U/mL) and absence of esterase. Notes: The conversion medium was phosphate-buffered saline (PBS, pH 7.4) supplemented with 0.5% (*w*/*v*) Tween 20 to ensure adequate solubilization of hydrophobic DEX-P. The data points represent the mean DEX conversion percentage, with error bars indicating the standard deviation of three independent experiments (mean ± SD, *n* = *3*).

The biotransformation of the lipophilic prodrug DEX-P into its active form (DEX) is a critical step for its therapeutic efficacy [53]. To evaluate stability and conversion kinetics, we monitored the hydrolysis of DEX-P in the presence and absence of esterase (Figure 6). In the control group without esterase (PBS, pH 7.4), no detectable conversion of DEX-P to DEX was observed throughout the 96 h incubation period, confirming the high chemical stability of the ester linkage under physiological conditions. In contrast, the addition of esterase triggered a gradual and time-dependent conversion. DEX conversion reached approximately 36.8 ± 6.4% at 24 h and 68.2 ± 7.6% at 48 h, eventually achieving nearly complete hydrolysis (99.8 ± 11.2%) by 96 h (Figure 6). These results indicate that DEX-P remains stable in physiological buffer but is efficiently activated biocatalytically in an enzyme-rich environment. As shown in Figure 6, a relatively high standard deviation was observed across the conversion profile, particularly at the later time points (72 and 96 h). This variation primarily stems from the low affinity of DEX-P toward hydrolyzing enzymes, combined with its gradual hydrolysis kinetics mediated by nonspecific carboxylesterases [53]. Nonetheless, the conversion profile in the presence of esterase supports that enzymatic hydrolysis occurs in a gradual and sustained manner.

This enzymatic conversion profile has significant implications for targeted inner ear delivery. Following IT administration, the DEX-P NS-TG resides in the middle ear cavity, where esterase activity in the middle ear fluid (effusion) is reported to be relatively low compared with that in serum [54]. The high stability of DEX-P in the absence of enzymes ensures that the drug remains primarily in its lipophilic prodrug form within the middle ear cavity [15,53,54]. Subsequently, the dissolved DEX-P molecules permeate the RWM as the prodrug form—a process significantly facilitated by the high lipophilicity inherent to the palmitate ester moiety. Upon crossing the RWM, the molecules encounter a substantially higher enzymatic environment within the cochlear tissues, triggering their conversion to active DEX. Previous studies have demonstrated that DEX-P can be gradually hydrolyzed to DEX by nonspecific carboxylesterases in biological matrices, including serum, liver, and macrophages [53], and have also reported general esterase activity in cochlear regions, including the stria vascularis and spiral ligament [55]. This localized abundance of esterase suggests that DEX-P is converted into active DEX upon permeating the RWM as the prodrug form.

### 3.6. In Vitro Cytotoxicity and In Vivo Biocompatibility Assessments

**Figure 7:**
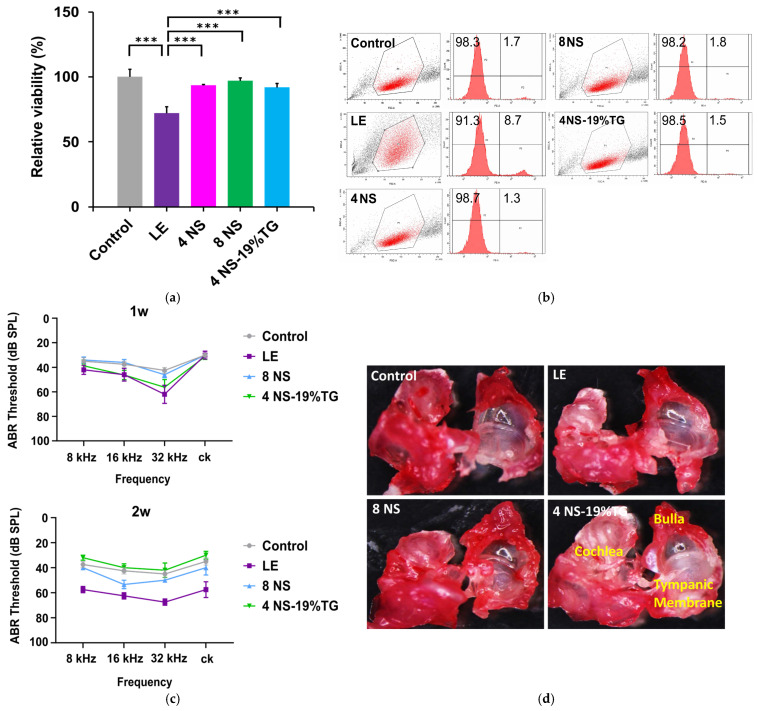
In vitro cytotoxicity and in vivo biocompatibility of DEX-P formulations in HEI-OC1 cells. (**a**) Relative cell viability measured by EZ-CytoX assay after 24 h of incubation. DEX-P LE served as a comparative control. All nanosuspension-based formulations (4 NS, 8 NS, and 4 NS–19%TG) showed no significant toxicity, whereas LE induced a marked reduction in viability (*p* < 0.001). Data represent the mean ± standard deviation (*n* = *3*). Statistical significance was defined as *p* < 0.05. *** indicates *p* < 0.001. (**b**) Representative flow cytometric analysis of PI-positive cells after treatment with each formulation for 24 h. The percentages shown in each panel indicate viable and dead cell populations. DEX-P NS formulations (4 NS, 8 NS, and 4 NS–19%TG) exhibited minimal membrane-compromised cell populations, comparable to those of the untreated control group, whereas LE treatment resulted in increased cell death. (**c**) ABR thresholds at 1 and 2 weeks following intratympanic administration of saline (Control), LE, 8 NS, or 4 NS–19%TG. No significant differences were observed among groups at 1 week, whereas only the control and LE groups showed a significant difference at 2 weeks. Data are presented as mean ± SD. ns, not significant; *p* < 0.05. (**d**) Representative macroscopic images of the tympanic membrane, bulla, and cochlea obtained after completion of the ABR assessment.

The in vitro cytotoxicity of the established DEX-P formulations (4 NS, 8 NS, and 4 NS–19%TG) was evaluated using HEI-OC1 cells, an immortalized mouse auditory cell line derived from the organ of Corti. This line is widely recognized as a specialized in vitro model for assessing ototoxicity and screening otoprotective agents, owing to its expression of characteristic markers for hair cells and supporting cells [30]. As shown in Figure 7a, the cell viability assay revealed a significant reduction in relative cell viability in the LE-treated group, which decreased to approximately 70% compared with the untreated control (*p* < 0.001). In contrast, 4 NS, 8 NS, and 4 NS–19%TG maintained high cell viability (>95%), with no statistically significant differences relative to the control group. This enhanced in vitro cytocompatibility was consistent with the FACS analysis (Figure 7b). The LE group exhibited a markedly higher proportion of PI-positive cells (8.7%) compared with the control (1.7%), whereas the 4 NS, 8 NS, and 4 NS–19%TG formulations demonstrated minimal PI-positive cell populations of 1.3%, 1.8%, and 1.5%, respectively. LE, which contains soybean oil and lecithin, can potentially trigger distinct toxicological pathways in sensitive auditory cells such as HEI-OC1. LE was previously reported to trigger cell death, accompanied by mitochondrial membrane depolarization and neutral lipid accumulation [56]. In addition, soybean oil is rich in ω-6 polyunsaturated fatty acids (PUFAs), notably linoleic acid [57]. High ω-6 levels have been shown to promote the generation of reactive oxygen species (ROS) and pro-inflammatory mediators, leading to mitochondrial dysfunction and subsequent apoptosis [58,59]. Furthermore, lecithin has been reported to pathologically increase membrane fluidity or disrupt lipid bilayer integrity, facilitating the leakage of intracellular components [60,61]. Considering that lipid vesicles are documented to internalize into cells via fusion or endocytosis [16,62,63], DEX-P LE likely facilitates rapid intracellular drug uptake through these pathways, thereby resulting in an acute toxic surge.

In contrast, the 4 NS, 8 NS, and 4 NS–19%TG formulations exhibited no noticeable cytotoxicity against HEI-OC1. This high biocompatibility is attributable to the use of inherently safe, FDA-approved stabilizers. PVA possesses a high safety threshold, with an oral LD50 in rats (approximately 15–20 g/kg) that is orders of magnitude higher than the concentrations used in this study [64]. Similarly, P407 is widely utilized in clinical IT and ophthalmic applications at concentrations up to 20–30 wt% without adverse effects [38,40,65]. Actually, to elucidate vehicle-induced toxicity, preliminary cytotoxicity evaluations of the blank NS and NS-TG vehicles were conducted, revealing no detectable toxicity. However, preparing an identical blank LE vehicle posed substantial technical constraints, as the stable fabrication of lipid emulsions requires highly specialized equipment, such as a high-pressure homogenizer, which was unavailable within the current experimental setup. Moreover, the “dissolution-limited” exposure profile of the NS systems compared with LE avoids the sudden intracellular dose surges associated with LE. Collectively, these findings suggest that the DEX-P NS and NS–TG systems offer a significantly safer therapeutic profile for IT administration, minimizing tissue damage within the delicate environment of the middle and inner ear.

To further evaluate the in vivo safety of the developed formulations, auditory function was assessed by ABR at 1 and 2 weeks following IT administration (Figure 7c). At 1 week, no significant differences in hearing thresholds were observed among the control, LE, 8 NS, and 4 NS–19%TG groups across all tested frequencies. At 2 weeks, the LE group exhibited significantly elevated ABR thresholds compared with the control group, whereas the 8 NS and 4 NS–19%TG groups remained comparable to the control. These findings indicate that the nanocrystal-based formulations did not induce measurable ototoxicity and exhibited a more favorable auditory safety profile than the LE formulation. Following the final ABR assessment, the bullae were opened and the tympanic membrane, middle-ear cavity, and cochlea were macroscopically examined under a surgical microscope (Figure 7d). Representative images demonstrated intact tympanic membranes without perforation or opacity and revealed no obvious signs of edema, hemorrhage, inflammatory exudate, or other gross pathological changes in the middle ear or cochlea. These findings suggest that both the 8 NS and 4 NS–19%TG formulations were well tolerated after IT administration and did not produce observable local tissue abnormalities under the present experimental conditions. Although microscopic histological evaluation would provide additional evidence of tissue compatibility, the combination of preserved auditory function and normal gross morphology supports the favorable in vivo safety profile of the developed formulations.

### 3.7. Storage Stability of Lyophilized DEX-P NS and NS-TG

The 4-week short-term stability study under ambient conditions (25 °C/60% RH) showed that the lyophilized DEX-P NS and DEX-P NS-TG were physiochemically stable for 4 weeks (Table 2). Visually, both the NS and NS-TG freeze-dried cakes maintained an elegant macroscopic structure without showing any signs of core collapse or hygroscopic shrinkage. Both NS and NS-TG formed homogenous suspension after the reconstitution process with water for injection. Following reconstitution, the mean particle size and zeta potential of the DEX-P NS-TG were determined to be 1163 ± 26.0 nm and −13.5 ± 0.9 mV, respectively, showing no marked alterations compared to the initial baseline values (0 weeks). The preservation of a narrow particle size distribution (PDI ≤ 0.30) indicates that the nanocrystals co-lyophilized with PVA and glucose were highly stable, effectively preventing detrimental phenomena such as irreversible nanocrystal aggregation, Ostwald ripening, or matrix-induced particle growth during both the freezing/thawing stresses and subsequent storage periods. Additionally, both DEX-P NS and NS-TG maintained their active drug content above 96%, denoting the absence of significant hydrolytic or oxidative degradation within the protected solid-state matrix. Nevertheless, comprehensive long-term (>12 months) and accelerated stability tests (at 40 °C/75% RH) are required to firmly establish the complete thermodynamic and chemical stability profiles of these novel formulations in subsequent investigational phases.

### 3.8. In Vivo Cochlear Drug Remaining Following IT Injection

**Figure 8:**
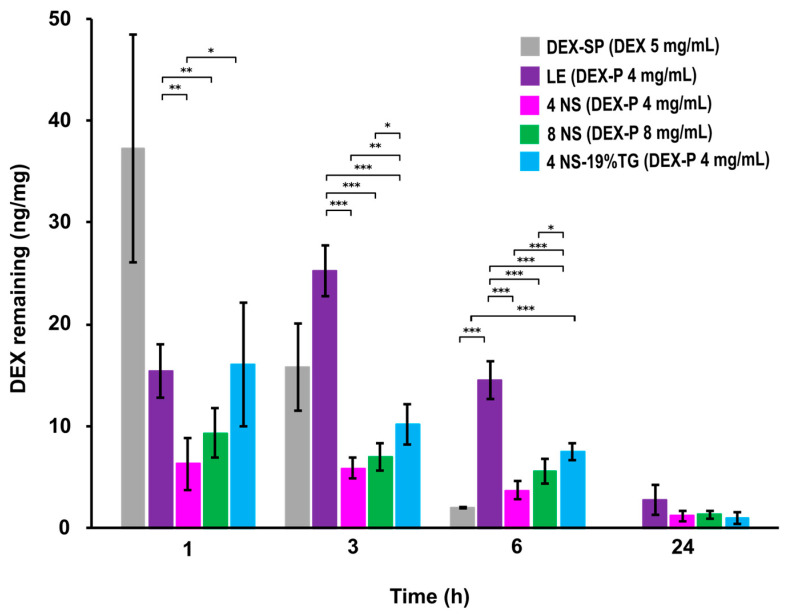
In vivo cochlear drug absorption following IT injection of DEX-SP solution, DEX-P LE, NSs and NS-TG. Drug concentrations (ng/mg) in cochlear tissues were measured at 1, 3, 6, and 24 h after a single IT administration of DEX-SP solution, DEX-P LE, 4 NS, 8 NS and 4 NS–19%TG. Notes: For in vivo IT administration, the DEX-SP solution was injected into the middle ear cavity at a volume of 5 μL, whereas the other formulations were administered at 10 μL as a single dose. The formulations included DEX-SP (DEX 5 mg/mL; total dose: 25 μg DEX), LE (DEX-P 4 mg/mL; total dose: 40 μg DEX-P, equivalent to 25 μg DEX), 4 NS (DEX-P 4 mg/mL; total dose: 40 μg DEX-P, equivalent to 25 μg DEX), 8 NS (DEX-P 8 mg/mL; total dose: 80 μg DEX-P, equivalent to 50 μg DEX), and 4 NS–19%TG (DEX-P 4 mg/mL; total dose: 40 μg DEX-P, equivalent to 25 μg DEX). Data are presented as mean ± SD (*n* = *5*). Statistical significance was defined as *p* < 0.05. Levels of significance are indicated as * *p* < 0.05, ** *p* < 0.01, and *** *p* < 0.001. Drug concentrations were quantified by LC-MS/MS after tissue homogenization.

The in vivo pharmacokinetic profiles of DEX in cochlear tissues were evaluated following IT injection to determine the delivery efficiency of the developed formulations (Figure 8). The simultaneous quantification of both the parent prodrug and DEX in cochlear tissues would offer a comprehensive understanding of the spatial distribution, transmembrane permeation, and bioconversion kinetics of our formulation. However, it is technically challenging to achieve the lower limit of quantification (LLOQ) required for the concurrent, reliable detection of both chemical entities via LC-MS/MS. Thus, we prioritized quantifying DEX in cochlear homogenates to validate the feasibility of the developed NS-TG system, as DEX level in the homogenates reflects the net outcome of DEX-P release, local solubilization, trans-RWM transport, and hydrolytic bioconversion. DEX-SP, a water-soluble prodrug solution, exhibited a rapid initial peak at 1 h, with a C_max_ of 37.5 ± 13.1 ng/mg (Table 3), but underwent a swift “burst-and-washout” pattern, with concentrations dropping to near-baseline levels by 6 h. This rapid clearance is a primary limitation of solution-based IT therapies, as the drug is prematurely eliminated through the Eustachian tube before sufficient transmucosal absorption [5,9].

In contrast, DEX-P LE showed a markedly enhanced and prolonged retention profile compared with DEX-SP. DEX-P LE achieved approximately 1.4-fold and 7.3-fold higher cochlear drug levels at 3 h and 6 h, respectively. Notably, DEX-P LE yielded a 2.3-fold increase in AUC_last_ relative to the DEX-SP solution (Table 3). Despite the constraints in achieving a precise comparative analysis due to the limited sampling time points, the AUC_last_ values provided meaningful insights into the local exposure of DEX following IT administration of each formulation. This high absorption efficiency is attributed to the solubilization of the lipophilic prodrug within the LE vehicle, which facilitates transport across the RWM. The RWM is a sophisticated trilayered semi-permeable barrier comprising an outer epithelium, a middle connective tissue layer, and an inner mesothelium [66,67]. While the tight junctions within the epithelial layer generally restrict the passive diffusion of high-molecular-weight or highly lipophilic molecules like DEX-P (630.87 Da), the LE facilitates penetration through several distinct mechanisms [10,66,67]. The high concentration of soybean oil and lecithin in the LE system functions as a potent chemical penetration enhancer [16,66]. The oil droplets in the dispersed phase provide a large contact surface area with the RWM, maintaining a high localized concentration gradient at the membrane interface, which drives the partitioning of the hydrophobic prodrug into the epithelial lipid bilayer. Furthermore, lecithin molecules can intercalate into the lipid bilayers of epithelial cells, increasing membrane fluidity and inducing a temporary, reversible disruption of tight junctional complexes [60,61]. This surfactant-mediated reduction in barrier resistance significantly enhances both paracellular and transcellular transport. Additionally, LE likely promotes internalization via lipid-mediated pathways, such as endocytosis or direct membrane fusion between oil droplets and apical cell membranes [62,63,68], allowing DEX-P to bypass the aqueous extracellular environment of the RWM and reach the perilymph more efficiently.

Conversely, the 4 NS and 8 NS exhibited relatively low cochlear concentrations, maintaining stable levels of 6.3 ± 2.6 ng/mg and 9.3 ± 2.4 ng/mg at 1 h, respectively (Figure 8). Despite the high dissolution rates observed in in vitro release profiles, their in vivo performance was likely hindered by the limited volume of inner ear fluids. In the restricted perilymphatic environment, the lack of a “sink condition” limits the dissolution of the crystalline drug, thereby reducing the concentration gradient required for RWM diffusion [50,69]. Interestingly, the 4 NS–19%TG system exhibited consistently superior cochlear delivery compared with NS formulations at all evaluated time points. At 1 h, 4 NS–19%TG achieved an initial absorption level of 16.1 ± 6.1 ng/mg, which was statistically comparable to that of LE (15.5 ± 2.6 ng/mg). This peak concentration was approximately 2.6-fold and 1.7-fold higher than those of 4 NS (6.3 ± 2.6 ng/mg) and 8 NS (9.3 ± 2.4 ng/mg), respectively. At 3 and 6 h, cochlear drug levels in the 4 NS–19%TG group (10.2 ± 1.9 and 7.5 ± 1.0 ng/mg, respectively) were lower than those of the LE group (22.4 ± 2.5 and 14.5 ± 1.8 ng/mg, respectively), yet remained significantly higher than the simple 4 NS and 8 NS groups (*p* < 0.05). Accordingly, as shown in Table 3, 4 NS–19%TG showed a C_max_ of 16.2 ± 5.9 ng/mg, which was 2.4- and 1.6-fold higher than those of 4 NS and 8 NS, respectively, while retaining approximately 64% of the C_max_ afforded by the LE system. It also achieved a 1.4-, 1.9-, and 1.3-fold higher AUC_last_ than the DEX-SP solution, 4 NS, and 8 NS, respectively, while retaining approximately 60% of the AUC_last_ afforded by the LE system. By 24 h, DEX concentrations in the 4 NS–19%TG group—similar to the LE group—declined to approximately 1.0 ng/mg, reflecting substantial clearance from the cochlea. At 48 h, drug levels were below the limit of detection. This observation aligns with prior reports on P407-based thermosensitive hydrogels, which, while functioning as localized depots, undergo gradual erosion and clearance via the Eustachian tube or mucosal absorption within 24–48 h in rodent models. The rapid turnover of middle ear secretions likely accelerates depletion of the gel reservoir, explaining the decline in drug levels by the 24 h mark [69,70]. The relatively rapid decline in drug concentration after 6 h, likely due to the erosion-prone nature of P407 in the dynamic middle ear environment, highlights the need for further formulation optimization to achieve multi-day sustained release [70,71]. Taken together, although NS–19%TG exhibited lower absorption than LE at 3 and 6 h, it represents a promising formulation approach by achieving a critical balance between delivery efficiency and biological safety.

In conclusion, the present study successfully fabricated a novel DEX-P NS TG formulation, providing its cytocompatibility, biocompatibility, and promising pharmacokinetic profiles through systematic in vitro and preliminary in vivo evaluations. Nevertheless, several critical checkpoints must be addressed in subsequent investigations to fully establish its clinical translatability. First, the therapeutic efficacy of this novel delivery system should be evaluated in disease-relevant hearing loss models, such as noise-induced trauma or cisplatin-induced ototoxicity. This will include functional recovery assessments via ABR and distortion product otoacoustic emission (DPOAE) threshold shift evaluations, alongside quantitative cochlear histopathology (e.g., outer hair cell survival analysis). Second, the in vivo biotransformation and permeation profiles require further elucidation. Simultaneous quantification of both DEX-P and DEX in ex vivo RWM setups or in vivo labyrinthine tissues using a validated LC-MS/MS assay is essential to provide precise insights into the transmembrane permeation mechanism and intra-cochlear bioconversion kinetics. In future studies, the sample size (currently *n* = *5* per group) will be expanded to minimize potential experimental variations, evaluate inter-animal variability, and verify statistical significance between the formulation groups. Third, repeated-dose toxicity studies remain essential to verify the long-term safety of the formulation upon IT administration. Finally, while the hydrogel system showed acceptable physicochemical stability for up to 4 weeks post-lyophilization, comprehensive long-term stability testing under different storage conditions is required to assess its clinical translatability.

## 4. Conclusions

In the present study, we successfully engineered an advanced, biocompatible nano-delivery platform for DEX-P for IT steroid therapy by integrating drug nanocrystallization with thermo-responsive hydrogel technology. The DEX-P nanocrystals, fabricated via a DC-based bead milling process, were stably incorporated into a 19% (*w*/*v*) P407-based matrix. This hybrid system not only significantly enhanced the apparent drug solubility through micellar solubilization but also ensured ease of administration followed by rapid in situ gelation at physiological temperatures. The novel system effectively mitigated the acute cytotoxicity in HEI-OC1 cells that is commonly associated with the commercial LE. Furthermore, in vivo pharmacokinetic evaluations in mice demonstrated that IT administration of 4 NS–19%TG achieved initial cochlear drug absorption levels comparable to those of LE and significantly higher than those of DEX-SP solutions and NSs. Collectively, our findings highlight the DEX-P NS–TG system as a safe and robust therapeutic platform for the localized treatment of sensorineural hearing loss and other inner ear disorders. Future research will focus on further refining the dissolution kinetics through particle size control and extending the residence time of NS–TG by enhancing the mechanical tenacity and mucoadhesive properties.

## Figures and Tables

**Table 1:** Physicochemical characteristics of DEX-P NSs and NS-TG.

Parameters	4 NS	8 NS	4 NS–19%TG
DEX-P conc. (mg/mL) *^(^^a)^*	3.86 ± 0.04	7.80 ± 0.09	3.89 ± 0.08
*Suspended* (mg/mL)	3.81 ± 0.04	7.72 ± 0.09	2.32 ± 0.08
*Dissolved * (mg/mL) * ^(a)^*	0.05 ± 0.00 *^(b)^*	0.08 ± 0.00 *^(b)^*	1.57 ± 0.02 *^(b)^*
Particle size (nm) *^(a)^*	835.0 ± 9.5 *^(c)^*	946.3 ± 10.9 *^(c)^*	1176.0 ± 11.0 *^(c)^*
PDI *^(a)^*	0.274 ± 0.033	0.281 ± 0.004	0.295 ± 0.018
Zeta potential (mV) *^(a)^*	−6.9 ± 0.7	−4.4 ± 0.1	−11.6 ± 0.9
Osmolarity (mOsmol/kg) *^(a)^*	319.3 ± 0.47	328.0 ± 0.82	300.0 ± 0.00
pH *^(a)^*	7.02 ± 0.01	7.00 ± 0.02	7.07 ± 0.01

*^(a)^* Data are represented as mean ± SD (*n* = *3*). *^(b)^* Values were calculated by subtracting the amount of dissolved drug from the total drug content in the formulation. *^(c)^* Mean hydrodynamic size was determined using dynamic light scattering (Zetasizer Nano^®^ Instruments).

**Table 2:** Physicochemical stability of DEX-P NS and NS-TG formulations after 4 weeks of storage at 25 °C/60% RH.

Parameters	4 NS	8 NS	4 NS–19%TG
DEX-P conc. (mg/mL) *^(^^a)^*	3.92 ± 0.07	7.72 ± 0.16	3.88 ± 0.11
Particle size (nm) *^(a)^*	856.15 ± 12.35	950.6 ± 20.3	1163 ± 26.0
PDI *^(a)^*	0.152 ± 0.044	0.190 ± 0.036	0.297 ± 0.036
Zeta potential (mV) *^(a)^*	−8.7 ± 1.2	−7.4 ± 0.5	−13.5 ± 0.9

Notes: All data were determined after reconstitution of the lyophilized samples with water for injection. *^(a)^* Data are represented as mean ± SD (*n* = *3*).

**Table 3:** Pharmacokinetic parameters of DEX in cochlear homogenates following IT administration of DEX-SP solution, DEX-P LE, DEX-P NSs, and DEX-P NS-TG.

Parameters	DEX-SP *	LE **	4 NS ^†^	8 NS ^††^	4 NS–19%TG ^‡^
C_max_ (ng/mg) *^(^^a)^*	37.5 ± 13.1 ^†,††,‡^	25.3 ± 2.5 ^†,††^	6.7 ± 2.1	10.2 ± 1.3	16.2 ± 5.9
T_max_ (h) *^(a)^*	1.0 ± 0.0	3.0 ± 0.0 *^,††,‡^	1.8 ± 1.0	1.4 ± 0.8	1.4 ± 0.8
AUC_last_ (ng·h/mg) *^(a)^*	98.9 ± 32.6	231.1 ± 64.2 *^,†,††,‡^	74.1 ± 15.5	101.6 ± 11.2	136.9 ± 16.4

*^(a)^* Data are represented as mean ± SD (*n* = *5*). Statistical significance was set at *p* < 0.05, with symbols denoting significant differences against the designated groups: * vs. DEX-SP, ** vs. LE, ^†^ vs. 4 NS, ^††^ vs. 8 NS, and ^‡^ vs. 4 NS–19%TG.

## Data Availability

The original contributions presented in the study are included in the article; further inquiries can be directed to the corresponding authors.
